# AI nutrition recommendation using a deep generative model and ChatGPT

**DOI:** 10.1038/s41598-024-65438-x

**Published:** 2024-06-25

**Authors:** Ilias Papastratis, Dimitrios Konstantinidis, Petros Daras, Kosmas Dimitropoulos

**Affiliations:** The Visual Computing Lab, Information Technologies Institute, Centre for Research and Technology Hellas, 57001 Thessaloniki, Greece

**Keywords:** Nutrition, Computer science

## Abstract

In recent years, major advances in artificial intelligence (AI) have led to the development of powerful AI systems for use in the field of nutrition in order to enhance personalized dietary recommendations and improve overall health and well-being. However, the lack of guidelines from nutritional experts has raised questions on the accuracy and trustworthiness of the nutritional advice provided by such AI systems. This paper aims to address this issue by introducing a novel AI-based nutrition recommendation method that leverages the speed and explainability of a deep generative network and the use of novel sophisticated loss functions to align the network with established nutritional guidelines. The use of a variational autoencoder to robustly model the anthropometric measurements and medical condition of users in a descriptive latent space, as well as the use of an optimizer to adjust meal quantities based on users’ energy requirements enable the proposed method to generate highly accurate, nutritious and personalized weekly meal plans. Coupled with the ability of ChatGPT to provide an unparalleled pool of meals from various cuisines, the proposed method can achieve increased meal variety, accuracy and generalization capabilities. Extensive experiments on 3000 virtual user profiles and 84000 daily meal plans, as well as 1000 real profiles and 7000 daily meal plans, demonstrate the exceptional accuracy of the proposed diet recommendation method in generating weekly meal plans that are appropriate for the users in terms of energy intake and nutritional requirements, as well as the easiness with which it can be integrated into future diet recommendation systems.

## Introduction

Over the past decade, AI has grown remarkably, giving rise to large deep networks and AI agents with impressive capabilities, even reaching human-level performance, in diverse domains. Such technological breakthroughs have unlocked significant opportunities, but also led to serious risks that include privacy violation, discrimination, as well as the ability of AI systems to achieve their objectives in ways that differs from the intended one^1–3^. In the field of nutrition specifically, AI systems have been widely proposed to provide personalized dietary advice. Nutrition plays a crucial role in adopting and maintaining a healthy lifestyle, while it also prevents the onset of serious non-communicable diseases (NCDs), such as obesity, cardiovascular diseases (CVD) and Type-2 diabetes (T2D)^4,5^. In addition, nutritious and balanced meals are regularly incorporated into treatment plans to alleviate the consequences or obstruct the further development of various diseases^6,7^. In this regard, AI systems that can automatically recommend personalized dietary meal plans can be immensely beneficial to the well-being of users. However, such AI systems face significant challenges that stem from the complexity of prioritizing actual needs of users^8^. Safety is also a crucial parameter in diet recommendations as unbalanced or harmful diets can lead to malnutrition. Such issues should be properly addressed for AI systems to be universally accepted as trustworthy diet recommenders.

Recently, the introduction of Large Language Models (LLMs) and more specifically of ChatGPT^9,10^, has sparked numerous discussions regarding its usage. Leveraging the low complexity, the high speed and an almost infinite pool of meals that it can draw from the web, ChatGPT can be used to make dietary recommendations to users^11^. However, an initial investigation of the safety and credibility of the provided meal recommendations unveiled that ChatGPT can be prone to errors^12^. On the other hand, traditional nutrition recommendation systems can achieve increased accuracy as they rely on experts’ knowledge and validated nutritional guidelines to provide highly balanced, nutritious and safe meal plans^13–16^. Despite the advantages, such systems suffer from reduced time efficiency and increased complexity due to the use of sophisticated ontologies and rules to filter inappropriate meals. Furthermore, the accuracy of traditional nutrition recommendation systems depends highly on the size and biases of the meal databases used to train them, limiting their applicability on certain population groups and thus their generalization capabilities.

In an effort to develop accurate and explainable AI-based nutrition recommendation systems, it is necessary to distill experts’ knowledge into these systems. To this end, this work proposes a novel AI-based diet recommendation system that can leverage the advantages of AI, such as speed, simplicity and generalization ability with the knowledge acquired from nutritional guidelines (i.e., European Food Safety Authority (EFSA)^17–19^ and World Health Organization (WHO)^20^) to guide the system towards accuracy and robustness. More specifically, the proposed system relies on a deep generative network and sophisticated loss functions to generate highly accurate personalized weekly meal plans in terms of energy intake and nutritional content through the modelling of user-specific information and the alignment of the network with well-defined nutritional rules, respectively. Moreover, leveraging on the ability of LLMs to produce equivalent meals, the proposed method significantly expands its meal database for improved accuracy and generalization ability. The contributions of this work are the following:

- A new deep generative network architecture is proposed to create weekly meal plans by employing sophisticated loss functions to align the network with well-founded nutritional guidelines.
- A novel approach for personalized nutrition recommendation that leverages the ability of LLMs to create an almost infinite pool of meals is proposed.
- The proposed AI-based diet recommendation system is validated on a large group of 3000 virtual user profiles and 1000 real profiles with 91000 daily meal plans, generated using meals from the Protein NAP database^21^ (large open-source collection of international meals), showcasing its advantages in terms of explainability and accuracy.

## Related work

**Figure 1:**
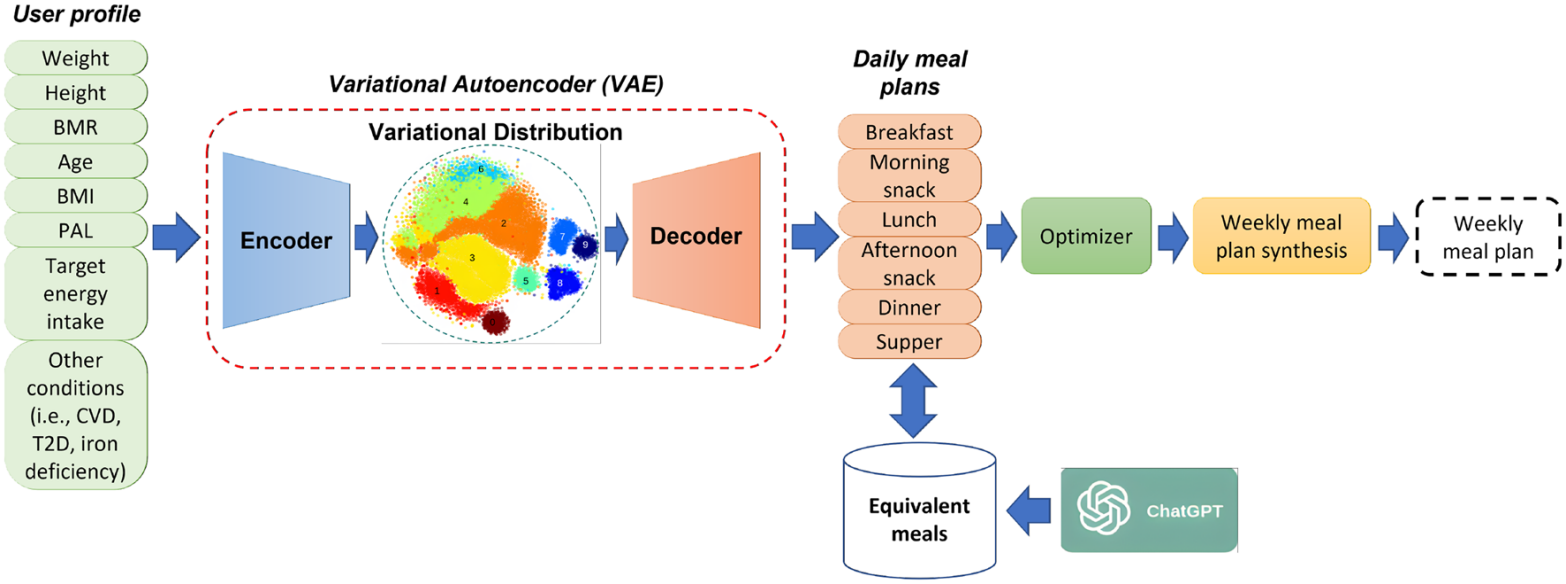
Overview of the proposed AI-based diet recommendation method.

In recent years, several works have been focused on the development of healthy food recommendations. In general, recommendation systems can be categorized as content-based (CB), collaborative filtering-based (CF), knowledge-based and deep-learning approaches. In order to provide individualized meal suggestions, content-based nutrition recommendation systems analyze the properties and composition of food products as well as the dietary requirements and preferences of users. Harvey et al. aimed to address inadequate nutrition by investigating participant perception of crucial aspects in recipes^22^. They created recommendation algorithms that take into account user preferences for ingredients, combinations and nutritional value. Teng et al. utilized ingredient networks (complement and substitute), which analyze nutritional data and web recipe collections to forecast recipe ratings^23^. This method reveals the connections between ingredients, consumer preferences and basic cooking principles. Gutiérrez et al. proposed an augmented-reality assistant that reads food products barcodes and provides recommendation, focusing on food quality and user preferences^24^. Shandilya et al. proposed a content-based recommender system that gives precedence to users’ recent preferences over previous ones, thus adapting to shifting user needs^13^. Their work focused on suggesting meals based on user preferences and dietary advice, ensuring that the daily nutrient needs are satisfied.

Collaborative filtering nutrition recommendation systems leverage user interactions and preferences to provide personalized dietary suggestions. These methods group individuals who have similar eating patterns and make meal recommendations based on user similarity. Ge et al. introduced a mobile app with a food recommendation system based on matrix factorization^25^. Their method models user preferences from user ratings and tags in order to offer personalized recipe suggestions. A different method for personalized nutrition recommendation was proposed by Yuan et al.^26^. This method initially employs k-means clustering to categorize foods into subsets prior to utilizing user-based collaborative filtering to suggest foods aligned with the user’s preferences and nutritional balance, considering standard recipes. Toledo et al. developed an approach for daily meal planning that incorporates both nutritional and preference factors^14^. It uses a sorting algorithm to filter the initial meal selection depending on user characteristics. Following that, an optimization phase creates a meal plan that prioritizes fewer recently consumed foods while aligning it with user preferences and nutritional requirements. On the other hand, user-based and item-based algorithms were tested for their ability to generate top-N suggestions by placing food items based on projected ratings^27^. The experimental results showed that the user-based algorithm outperforms the item-based one in terms of accuracy and coverage. Finally, Rostami et al. proposed a two-phase food recommendation system, which starts by using time-aware collaborative filtering to suggest food items based on previous user consumption and then it forecasts food item ratings using nutritional information^15^. To group comparable people and food products, the system uses a clustering method, which improves recommendation accuracy.

Knowledge-based nutrition recommender systems use specialized knowledge of nutrition and dietary recommendations to give consumers personalized nutritional advice. These systems combine expert knowledge in addition to simple data-driven methods to provide highly individualized meal plans. A personalized knowledge-based dietary recommendation method targeting obese users was proposed by Jung and Chung^28^. Their method uses collaborative filtering along with knowledge-based context data to create individualized diet menus. By using context-aware modeling, it also addresses the problem of sparse data and enables mobile users to access tailored menus and recipes for the treatment of obesity. Similarly, Mckensy-Sambola et al. adopted a knowledge-based diet recommendation system that employs user data to deduce the best diets and then provides a list of recipes that satisfy the user’s nutritional needs^29^. The Ontology of Dietary Recommendations (ODR) is used to get the most important components in the domain of food recommendations, such as diets, recipes, ingredients, anthropometric indices and food allergies. Another research work employed a collaborative recipe knowledge graph (CRKG) that incorporates user interactions and food-related data to develop a novel food recommendation model^30^. The health-aware food recommendation model utilizes a healthiness matching score and a knowledge-aware attention graph convolutional neural network to combine user preferences and health requirements and generate healthy personalized recommendations. Recently, a knowledge-based recommendation framework for precise diet planning across various user groups, including both healthy individuals and those with health conditions, was introduced^16^. The framework comprises two key layers: a qualitative layer employing expert-derived rules and an ontology^31^ for ingredient validation and a quantitative layer utilizing optimization techniques to create daily meal plans based on specific nutrient requirements. Extensive experiments demonstrated the system’s effectiveness in generating appropriate, diverse and tailored meal plans.

Nowadays, deep learning methods have achieved outstanding performance in several research areas including nutrition recommendation. Mokdara et al. proposed a food recommendation system that integrates deep neural networks (DNNs) with user-provided favorite ingredients^32^. Then, a temporal prediction model forecasts future dish recommendations based on the user’s profile and eating habits. Another work proposed an explainable food recommendation system that is based on deep image clustering to incorporate visual content to improve nutrition recommendations^33^. Moreover, a similarity score is adopted to propose foods that match with the user preferences. Iwendi et al. proposed to utilize several deep learning techniques for diet recommendation based on patients’ characteristics, such as disease, age, weight and gender^34^. The method was tested on medical datasets from hospitals and it was found that LSTM was the best technique, since it modelled better the medical history. Finally, Zhang et al. proposed a method for sequence-based recommendations using dynamic user-item interactions^35^. More specifically, a Long Short-Term Memory (LSTM) network was employed to capture sequential information and used collaborative filtering to suggest personalized meal plans. However, most existing nutrition recommendation systems lack personalization and provide more generic recommendation that may not be suitable for everyone.

To overcome the limitations of current nutrition recommendation systems, several studies leveraged the advancements achieved by LLMs in several fields for personalized nutrition recommendation. Niszczota et al.^12^ used ChatGPT to create diets and accommodate specific food allergies. The authors found out that ChatGPT could produce balanced diets but there were inaccuracies in the proposed diets with respect to food calories and meal portions. Tsai et al.^36^ used ChatGPT to provide tailored pregnancy nutrition advice for underserved populations. This method aimed to address health disparities and adverse outcomes linked to low socioeconomic status and inadequate nutrition during pregnancy. Other studies used ChatGPT diet recommendations for diabetes and obesity treatments and stressed out the generic nature of the proposed meals, the limited understanding of context and privacy and security concerns associated with the use of LLMs^11,37^.

This work proposes a deep generative network to create accurate weekly meal plans by leveraging both the speed, simplicity and generalization ability of AI and the nutritional experts’ knowledge in the form of guidelines. Through the use of novel loss functions that implement the nutritional guidelines and the ability of ChatGPT to create a large meal database, the proposed AI-based diet recommendation system can provide accurate nutritional advice aligned with expert validated rules.

**Figure 2:**
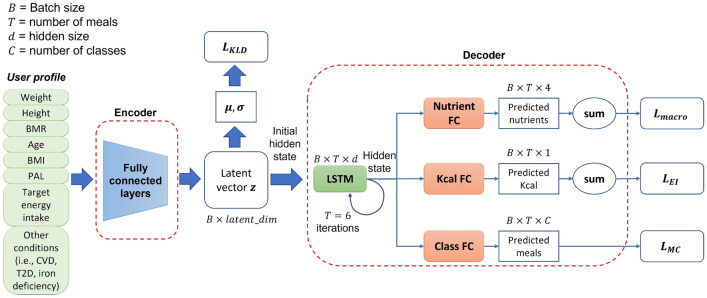
Internal architecture of the variational autoencoder.

## Method

The proposed AI-based diet recommendation method utilizes a novel deep generative network architecture to provide personalized meal plans to users based on their profile. More specifically, a variational autoencoder network processes the input, which is a vector that contains individual information (e.g., weight, height, age, etc.). The produced feature representation lies in a latent space, in which the input information can be modelled in an optimal way and capture meaningful and informative features about the user’s dietary requirements. Subsequently, a recurrent neural network is utilized to generate sequences of meals and construct the weekly meal plan. At the last stage, the generated meal plans are fed to an optimizer that adjusts the meal quantities to ensure that the energy and nutrients align with the user’s requirements and a final weekly meal plan is formulated. Additionally, ChatGPT^10^, a powerful language model developed by OpenAI, is adopted in order to expand the original meal database and enhance meal recommendations. The overview of this method is depicted in Fig. 1, while the main components of the proposed architecture are described in detail in the following subsections.

The user profile comprises the only input to the proposed AI-based diet recommendation method and it is crucial for the creation of valid personalized nutritional advice that adheres to user’s needs and nutritional guidelines. The user profile holds important anthropometric measurements and information on the physical activity status and medical condition of a user. More specifically, the profile consists of the weight, the height, the basal metabolic rate (BMR), the age, the body mass index (BMI), the targeted energy intake, the physical activity level (PAL) and the existence or not of cardiovascular disease, type-2 diabetes (T2D) and iron deficiency. These values are normalized and aggregated to form a feature vector that describes the user and is fed as input to the variational autoencoder network.

### Variational autoencoder

The variational autoencoder (VAE) network is a core part of the proposed AI-based diet recommendation method and comprises two main components, namely the encoder and the decoder. In the context of meal plan recommendation, the goal of the VAE network is to develop a probabilistic generative model of data and transform the input (i.e., user profile) into a more powerful and discriminative feature representation that lies in a latent space. The new latent space representation is capable of capturing meaningful and informative features about the user’s dietary requirements, which can then be used to generate personalized meal plans. The use of the latent space to model the input contributes significantly to the explainability of the proposed method as it allows the formation of clusters of users with similar nutritional needs. The architecture of the VAE is depicted in Fig. 2.

The primary function of the encoder is to map the input vector (i.e., user profile) into a new more powerful and discriminative feature representation that lies in a latent space. This step is critical as the quality of the representation in the latent space directly impacts the efficacy of the generated meal plans. Given the user profile vector $\textbf{x} = (x_1, x_2, \dots , x_u) \in \textrm{R}^u$, the encoder employs a fully connected layer to map the vector into a new feature vector $\mathbf {x'} = f(\textbf{x})$. Then, the new feature vector $\mathbf {x'}$ is mapped into a compact and structured representation, i.e., the latent space. To construct this latent space of the user profiles, two fully connected layers are employed to generate the mean $\mu = f_{\mathbf {\varvec{\mu }}}(\mathbf {x'}) ,\mu \in \textrm{R}^d$ (where *d* is the size of the latent space) and the standard deviation $\log ({\varvec{\sigma }}^{2}) = f_{\sigma }(\mathbf {x'}) , \sigma \in \textrm{R}^d$ vectors of the multivariate Gaussian distribution from the feature vector $\mathbf {x'}$, where $f_{\mu }$ and $f_{\sigma }$ denote the fully connected layers responsible for computing the mean and standard deviation vectors of a multivariate Gaussian distribution, respectively. This distribution comprises the latent space that can better model the input user profile vectors. As a result, each dimension in the latent space effectively captures changes in the users’ nutritional needs, dietary requirements and overall health. In addition, in the latent space, users with similar profiles and thus similar nutritional needs are closer to each other and far away from users with significantly different profiles. Such a modelling enables the proposed AI method to improve its explainability by justifying its decisions through a collaborative filtering mechanism (i.e., similar users are provided with similar recommendations).

Through the well-known reparameterization trick^38^, a latent vector $\textbf{z} = \mu + \sigma \odot \varepsilon$ is computed from the multivariate Gaussian distribution $\mathcal {N}(\mu ,\sigma )$, where $\varepsilon \sim \mathcal {N}(0,1)$ is a random value sampled from a standard Gaussian distribution. Once the descriptive latent representation of the input has been created via the encoder, the responsibility of the decoder is to transform the latent vector $\textbf{z}$ into a personalized daily meal plan. The use of recurrent neural networks is motivated by its proven ability to handle sequences efficiently, making it ideal for generating a series of 6 meals for a single day (i.e., breakfast, morning snack, lunch, afternoon snack, dinner and supper). To this end, the decoder uses the Gated Recurrent Unit (GRU) module to handle the generation of a sequence of meals efficiently. The GRU takes as input the latent vector $\textbf{z}$ and generates the daily meal plan $\hat{\textbf{y}}({t}), ~t=1\dots 6$ as follows:

1 $$\begin{aligned} \textbf{h}(t)&= {\left\{ \begin{array}{ll} \text {GRU}(\textbf{z}) &{} \text {for } t=1 \\ \text {GRU}(\textbf{h}({t-1})) &{} \text {for } t>1 \end{array}\right. } \end{aligned}$$

where $\textbf{h}(t)$ is the hidden state at time *t*, considering the meals as a temporal sequence spanning from breakfast to supper. The first input to the GRU at time $t=1$ is the latent vector $\textbf{z}$, while for the next timesteps (i.e., $t>1$) the previous hidden state $\textbf{h}({t-1})$ is fed to the GRU. The predicted meal classes $\hat{\textbf{y}(t)}$ are finally computed as:

2 $$\begin{aligned} \textbf{o}(t)&= \text {softmax}(f_m(\textbf{h}(t))),~~ t=[1,6] \end{aligned}$$

3 $$\begin{aligned} \hat{\textbf{y}({t})}&= \arg \max _c(\textbf{o}(t)),~~~~~~~~~~ t=[1,6] \end{aligned}$$

where $f_m$ is the classifier (i.e., fully connected layer) and $\mathbf {o(t)}$ is the output class probabilities at meal time *t*. The most probable class for a meal is identified by a $\max$ operation on all class probabilities *c* as shown in Eq. 3. Two additional fully connected layers are used to predict the total energy $\hat{EI}$ and the nutrient values $\hat{n}$ of the predicted daily meal plan, which are computed as:

4 $$\begin{aligned} \hat{EI}&= \sum _{t=1}^{T}f_{EI}(\textbf{h}(t)) \end{aligned}$$

5 $$\begin{aligned} \hat{n}&= \sum _{t=1}^{T} f_{nutr}(\textbf{h}(t)) \end{aligned}$$

where $f_{EI}$ is the fully connected layer that predicts the calories of the meal plan and $f_{nutr}$ predicts the macronutrients of the meal plan.

### User profile

The user profile comprises the only input to the proposed AI-based diet recommendation method and it is crucial for the creation of valid personalized nutritional advice that adheres to user’s needs and nutritional guidelines. The user profile holds important anthropometric measurements and information on the physical activity status and medical condition of a user. More specifically, the profile consists of the weight, the height, the basal metabolic rate (BMR), the age, the body mass index (BMI), the targeted energy intake, the physical activity level (PAL) and the existence or not of cardiovascular disease, type-2 diabetes (T2D) and iron deficiency. These values are normalized and aggregated to form a feature vector that describes the user and is fed as input to the variational autoencoder network.

### Loss functions

A novel set of loss functions are proposed to guide the training of the proposed network towards recommending accurate and personalized meal plans. More specifically, the loss functions aim to penalize deviations of the proposed meal plans from the nutritional requirements (i.e., energy and macronutrient intake) defined by the well-established EFSA^17–19^ and WHO^20^ guidelines for the specific user profile. In this way, the proposed deep network is guided towards providing personalized nutritional advice that satisfy the user needs based on their anthropometric features and medical conditions, thus aligning the proposed AI-based diet recommendation system with the principles of AI trustworthiness.

At first, a macronutrient penalty loss $L_{\text {macro}}$ is used to minimize the difference between the nutritional content of the predicted meal plan and the nutritional rules and guidelines.

6 $$\begin{aligned} L_{\text {macro}} = \frac{1}{N}\sum _{i=1}^{N} \left( |min\_val(i)- \hat{n})| + |max\_val(i) - \hat{n}|\right) , \end{aligned}$$

In Eq. 6, *N* is the number of macronutrients (i.e., protein, carbohydrates, fat and saturated fat acids (SFA) and $min\_val(i)$ and $max\_val(i)$ is the minimum and maximum suggested values for each nutrient *i*, since in the nutritional rules the recommended macronutrient intake is defined in ranges. The goal of the macronutrient penalty loss is to align the meal plan proposed by the AI-based diet recommendation system with the nutritional requirements of the user based on their profile. A second loss term, named energy intake loss $L_{EI}$, is also proposed to calculate deviations, in the form of mean squared error, between the predicted caloric energy intake $\hat{EI}$ of the recommended meal plan and the appropriate energy intake *EI* based on the user profile.

7 $$\begin{aligned} L_{\text {EI}} = \frac{1}{N} \sum _{i=1}^{N} (EI - \hat{EI})^2 \end{aligned}$$

where the personalized target daily energy intake is calculated based on the factorial approach for computing total energy expenditure^39^ as:

8 $$\begin{aligned} EI = \left\{ \begin{array}{lr} BMR*PAL+D, &{} BMI\le 18.5\\ BMR*PAL, &{} 18.5< BMI < 25\\ BMR*PAL-D,&{} 25\le BMI \end{array}\right\} \end{aligned}$$

In Eq. 8, we consider a parameter *D* that is used to increase or decrease the target energy intake and assist users with too small or too high BMI achieve the goal of gaining or losing weight. The goal of the energy intake loss is to guide the proposed AI-based diet recommendation method towards suggesting accurate meal plans that satisfy a user’s energy requirements. The losses in Eqs. 6, 7 ensure that the computed meal plans are of high accuracy and comply with the nutritional and energy intake guidelines.

Apart from the aforementioned loss functions, there are two additional losses that are employed to guide the network towards the better modelling of the input space and the compliance with ground truth labels (i.e., meals), respectively. The Kullback–Leibler Divergence (KLD) is employed in order to train the encoder to produce a distribution in the latent space that closely matches a desired distribution, typically a standard Gaussian. This is done to ensure that the latent space is well-structured and can be effectively sampled during the generative phase of the VAE for a user profile. This loss is computed as:

9 $$\begin{aligned} L_{\text {KLD}} = -\frac{1}{2} \sum _{j=1}^{J} (1 + \log ((\sigma _j)^2) - (\mu _j)^2 - (\sigma _j)^2) \end{aligned}$$

where *J* is the total number of dimensions in the latent space, $\sigma _j$ represents the standard deviation of the *j*-th dimension of the learned latent variable distribution and $\mu _j$ represents the mean of the *j*-th dimension of the learned latent variable distribution. $L_{\text {KLD}}$ aims to distribute all latent space encodings for all inputs evenly around the center of the latent space. In this way, the embeddings are as close as possible to each other while still being distinct, allowing smooth interpolation and enabling the generation of new samples.

The last loss function is the cross entropy loss $L_{MC}$ that calculates the differences between the output class probabilities and the target meal classes that are provided by the training dataset. This loss function ensures that the meal classifier predicts personalized meals close to the ground truth data and is calculated as:

10 $$\begin{aligned} L_{MC} = -\sum _{t=1}^{T=6}\left( \sum _{c=1}^My_{o_t,c}\log (p_{o_t,c})\right) \end{aligned}$$

where *M* is the total number of different meals, $y_{o_t,c}$ indicates whether the class label *c* is the correct classification for observation $o_t$ and $p_{o_t,c}$ is the probability of class *c* for the output $o_t$.

Finally, the total loss *L* for the proposed diet recommendation method is defined as:

11 $$\begin{aligned} L = L_{MC} + L_{\text {KLD}} + L_{\text {EI}} + L_{\text {macro}} \end{aligned}$$

The final loss function aims to structure the latent space in such a way that allows similar user profiles to be close to each other in the latent space, while dissimilar user profiles to be located far from each other, thus enabling the formation of user groups or clusters. In addition, these losses contribute significantly to the explainability of the proposed diet recommendation method by clarifying why two users have been provided with similar or totally different meal plans.

**Figure 3:**
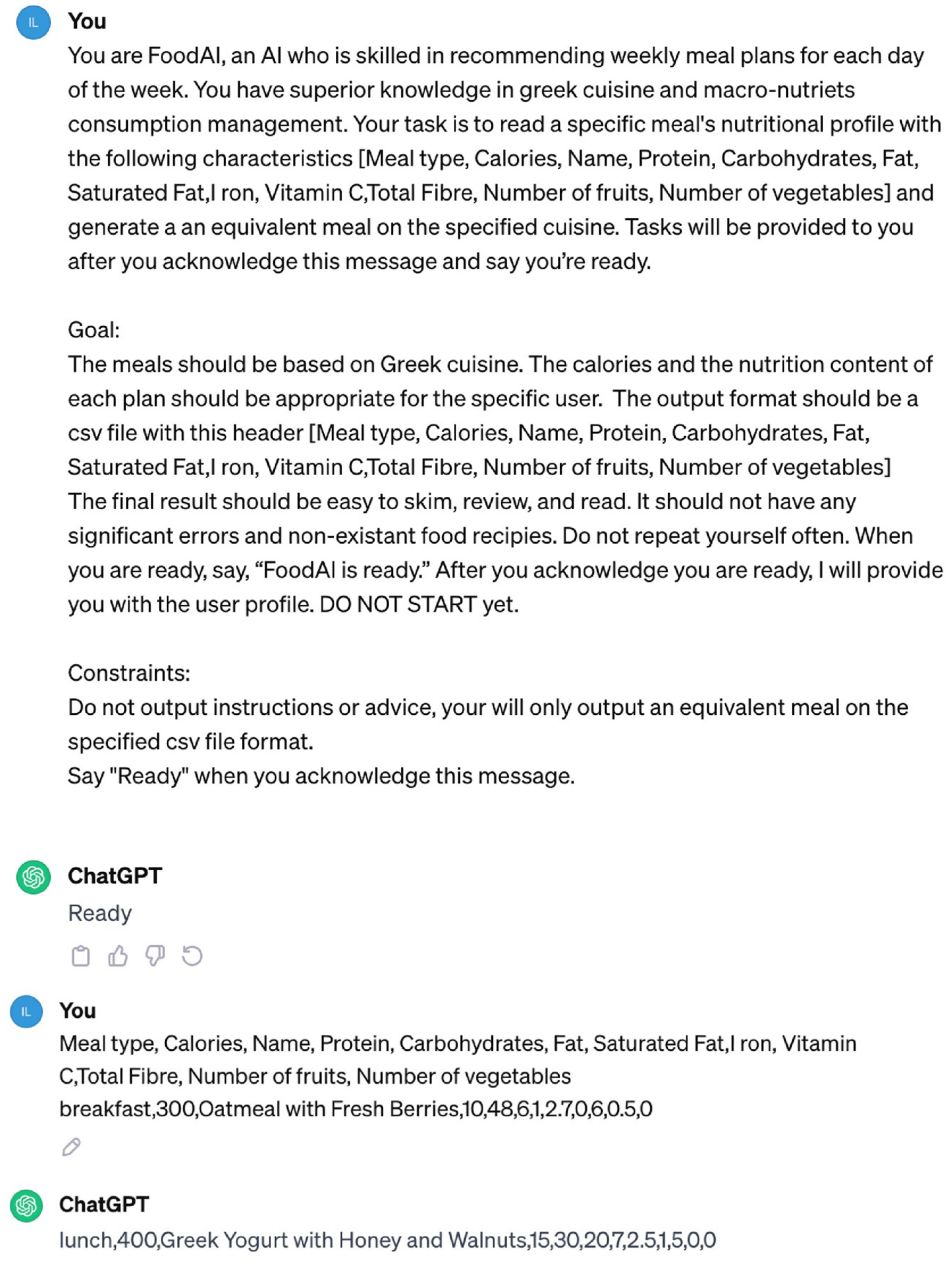
Example of the equivalent meal generation process from ChatGPT.

### Meal plan generation

For the creation of the daily meal plan, the decoder runs for 6 timesteps to generate 6 daily meals from each category (i.e., breakfast, morning snack, lunch, afternoon snack, dinner and supper). This output represents the probabilities assigned by the decoder for each meal type and for each meal category and the meal type with the highest probability is selected as the optimal one for the corresponding meal category, according to Eqs. 2, 3. For generating a weekly meal plan, the aforementioned process of daily meal plan generation is repeated 7 times (i.e., one daily meal plan for each day of the week) by sampling different latent vectors from the variational distribution. Moreover, a masking strategy is employed to the output probabilities to ensure meal variation and prevent the VAE from producing similar meal plans every day. The goal of this masking procedure is to keep track of meals selected in previous days of the week and achieve meal diversity. An increased meal diversity can make a meal plan more balanced through the inclusion of various food groups and more attractive and enjoyable for the user, thus increasing the adherence of the user to the recommended meal plan.

### Optimizer

The optimizer is the final component of the proposed AI-based diet recommendation method that aims to ensure that the energy intake of the recommended daily meal plan satisfies the user’s energy requirements. This component enhances the accuracy of the proposed method since mismatched energy intake can lead to a plethora of health issues, from weight gain to malnutrition. The optimizer layer acts as a regulatory system, making real-time adjustments to the meal portions based on the targeted energy intake *EI*. The goal of the optimizer is to match the energy intake of the proposed meal plan $\hat{EI}$ to the targeted energy intake of the user. To achieve this, the optimizer changes the meals portions *mp* in order to zero out the caloric difference of the energy intake of the predicted meal plan and the targeted energy intake of the user. This is done by calculating the percentage of the difference *d* as follows:

12 $$\begin{aligned} d = \frac{EI - \hat{EI}}{\hat{EI}} \end{aligned}$$

Finally, the new meal portions ${mp}'$ are changed as:

13 $$\begin{aligned} mp' = d*mp+mp \end{aligned}$$

This adjustment ensures that the users consume appropriate meal quantities that correspond to the targeted energy intake and macronutrients of the users based on their personal characteristics and health condition, thus improving the accuracy of the proposed diet recommendation method.

### Equivalent meals with ChatGPT

Current diet recommendation systems face an inherent limitation of relying on meal databases with a finite set of meals, meals with incomplete nutritional information or meals generated for specific populations for their training and evaluation^40,41^. As a result, such systems demonstrate limited ability in providing accurate, reliable and diverse personalized dietary advice. To overcome the aforementioned limitation of diet recommendation systems, this work adopts ChatGPT to expand the meal database of the proposed method in a way that is no longer restricted by its size or the user group that is applied to. In this way, the proposed method leverages the ability of LLMs to acquire information from the web in order to build a large meal database for performing highly accurate meal recommendations for different population groups.

To achieve this, ChatGPT is utilized and instructed to generate meals with similar nutritional profiles and calories. More specifically, an initial standardized query is fed to ChatGPT, which describes the process and instructs it to acknowledge this specific task. By presenting ChatGPT as “FoodAI,” an AI system that is proficient at recommending meals and knowledgeable about many world cuisines, the equivalent meal recommendation process is initiated. ChatGPT is taught to acknowledge the task by saying, “FoodAI is ready”, making sure it is aware of the objectives and limitations before moving forward. Then for every meal in our database, a query with the meal ingredients and nutrition facts is fed to the model. Finally, ChatGPT scans its knowledge base to find dishes from these cuisines that share similarities in ingredients, nutrition facts and calories with the queried meal. The returned meals are parsed and mapped to our meal structure, ensuring compatibility with the rest of our recommendation system. The output is required to be structured like a Comma Separated Values (CSV) file and includes the following headers: Meal name, Meal type, Calories, Protein, Carbohydrates, Fat, Saturated Fat, Iron, Vitamin C, Fibre, Number of fruits and Number of vegetables. An example of this query is given in Fig. 3.

**Figure 4:**
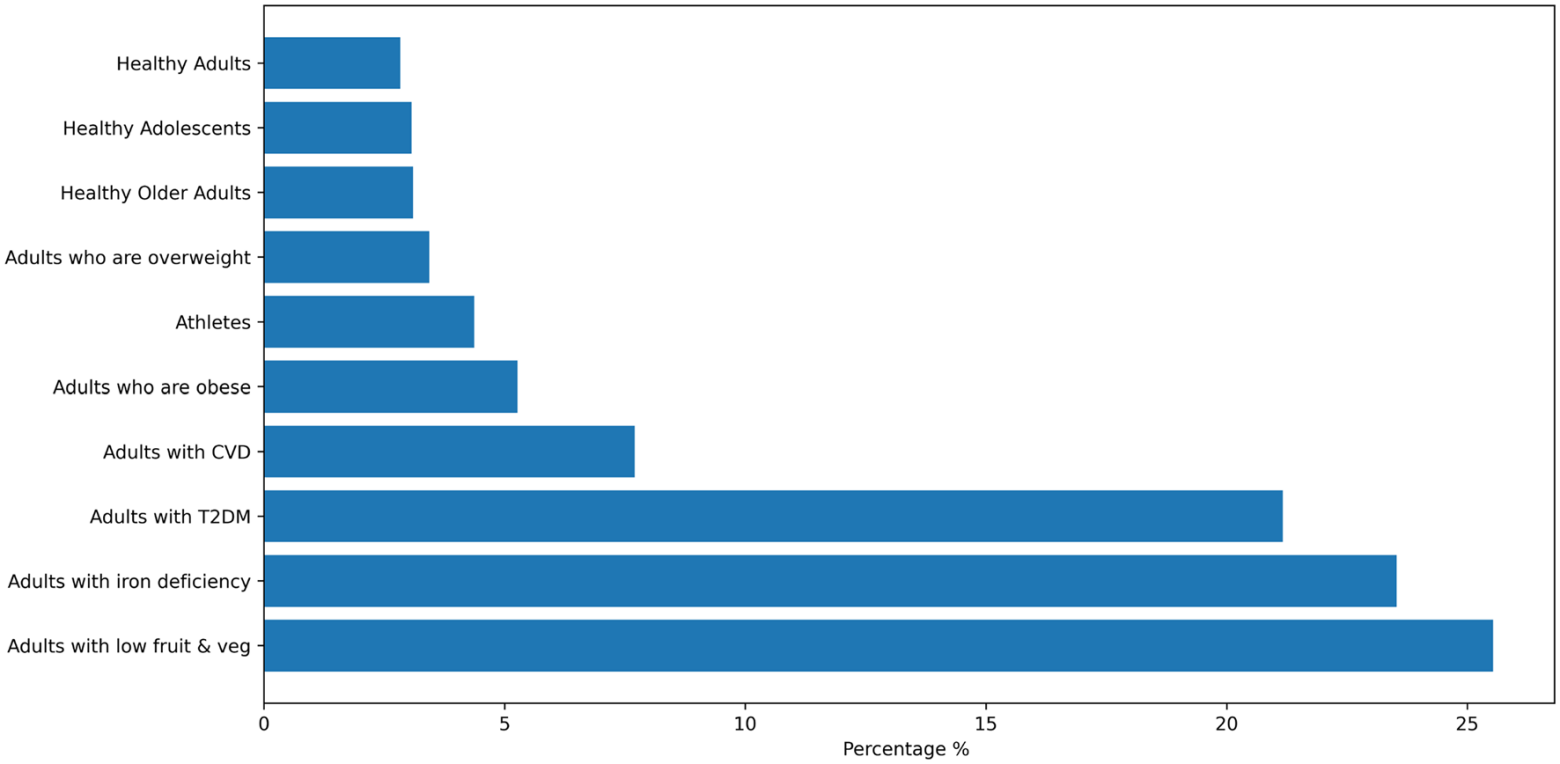
Distribution of 3000 virtual user profiles per user group.

## Experimental results

### Datasets

**Table 1:** Caloric and nutrient content of the unique meal types in the 84000 meal plans, depicted in mean ± std format.

Meal type	Calories (kcal)	Protein (g)	Carbohydrates (g)	Fat (g)	SFA (g)
Breakfast	423.75 ± 157.87	20.07 ± 9.45	56.95 ± 23.50	14.53 ± 9.49	4.24 ± 3.31
Morning snack	169.16 ± 136.85	7.03 ± 8.11	27.22 ± 22.11	4.45 ± 5.84	1.28 ± 2.20
Lunch	543.52 ± 166.30	34.85 ± 16.63	60.88 ± 23.51	18.37 ± 9.46	4.64 ± 3.77
Afternoon snack	211.94 ± 144.87	8.36 ± 8.22	31.67 ± 22.73	6.67 ± 7.07	2.07 ± 2.83
Dinner	600.19 ± 259.45	37.53 ± 19.39	75.58 ± 49.71	21.65 ± 12.38	6.38 ± 4.89
Supper	189.93 ± 103.24	10.70 ± 7.65	21.21 ± 15.30	7.35 ± 6.60	2.09 ± 2.07

In this work, we use the Protein NAP database^21^, which is an open-source dataset of Nutrition and Physical Activity Plans (NAPs) that has been approved by nutritional experts and physicians, for meal plan generation. It contains foods, meals recipes and physical activities constructed from nutritionists, doctors and other medical professionals in accordance with WHO^20^ and EFSA^17–19^ guidelines. To train the proposed method, we use 84,000 daily meal plans that have been created for 3000 user profiles (i.e., 3000 users multiplied by 7 days by 4 weeks) following the approach proposed by Stefanidis et al.^16,42^, where 80% of the plans is used for training and 20% for testing. These profiles reflect a representative user population, since the demographic data (gender, age, height, weight and health issues) have been computed using either normal or uniform distributions. More specifically, users are equally split between men and women, their age is between 20–88 years old, the weight is in the range of 46–180 kilograms and the height is in the range of 1.60–2.0 meters. Regarding the health condition, the user profiles are distributed across 10 categories, being healthy users (i.e., adults ($N = 85$), adolescents ($N=158$, older adults ($N=131$), athletes ($N=103$), adults who are overweight ($N=92$, adults who are obese ($N=635$) and adults with cardiovascular disease (CVD) ($N=706$), Type-2 Diabetes ($N=766$), iron deficiency ($N=231$) or low in fruits and vegetables ($N=93$). A visualization of the user profiles’ distribution per user group is shown in Fig. 4. Each meal plan consists of 6 meal types consumed during a day, namely, breakfast, morning snack, lunch, afternoon snack, dinner and supper. The total number of unique meals is 1349 and for each meal type, there are 286 breakfasts, 258 morning snacks, 255 lunches, 253 afternoon snacks, 238 dinners and 59 suppers, respectively. Statistics regarding the average calories and nutrients of the unique meal types contained in the 84000 meal plans are shown in Table 1.

To further validate the performance of the proposed AI-based diet recommendation system, experiments were performed with real profiles that correspond to human subjects participating in two different studies. The data from these studies are available in two open datasets^43,44^. The first dataset was created for the estimation of obesity levels based on eating habits and physical condition^43^. From this dataset, 250 individuals with normal weight, 250 individuals classified as Overweight level I or II and 250 individuals classified with Obesity level I, II or III were randomly extracted. On the other hand, the second dataset contains profiles of patients with cardiovascular diseases^44^. From this dataset, 250 individuals with cardiovascular diseases and no other comorbidities were randomly selected. As a result, a total of 1000 real profiles were collected that are equally distributed among 4 different user groups (Healthy, Overweight and Obese adults, and adults with CVD), similar to the user groups that were used for the training of the proposed AI-based diet recommendation system. This set of 1000 real profiles was used only for the evaluation of the trained diet recommendation system.

**Figure 5:**
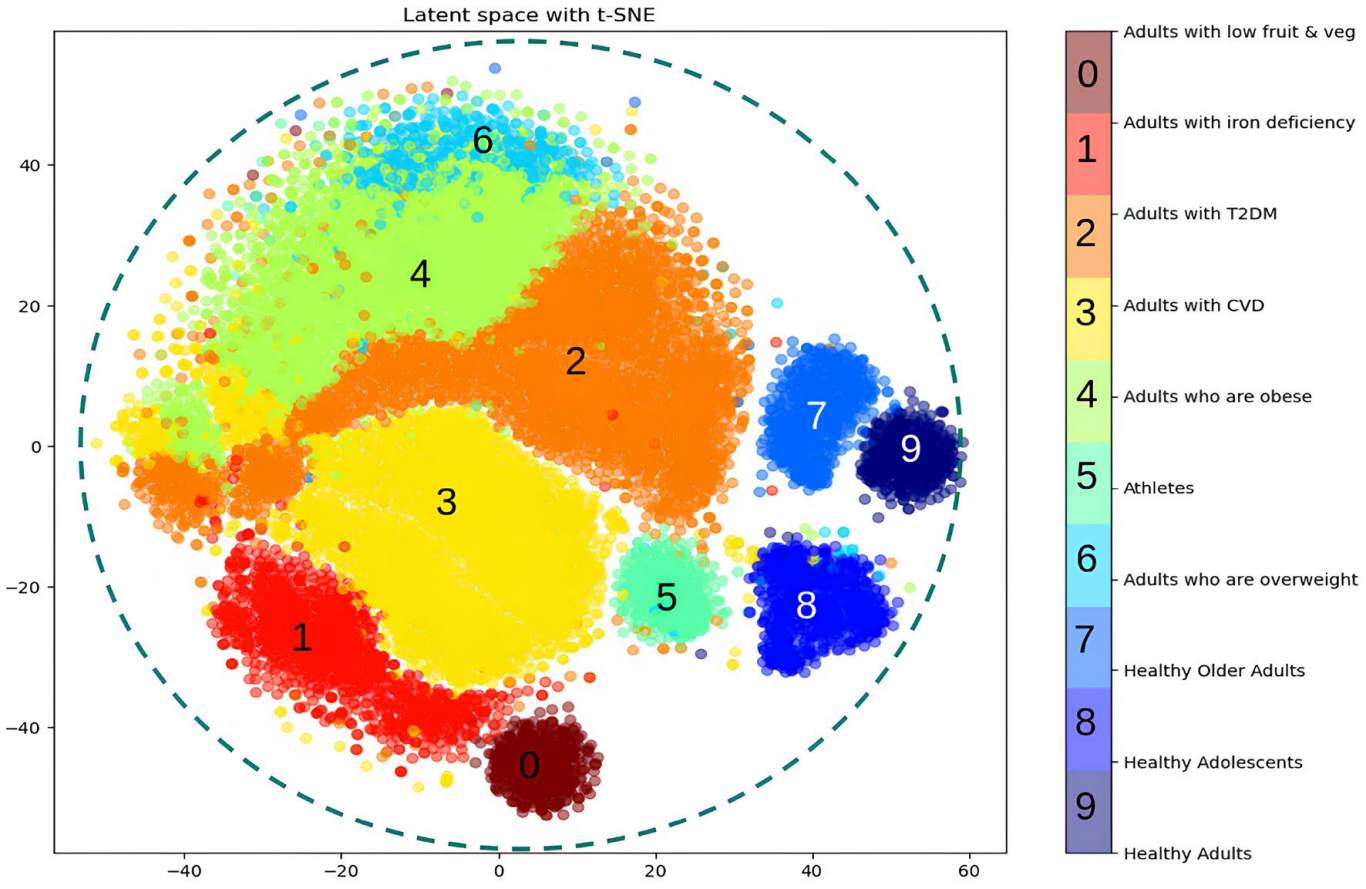
Visualization of the latent space for 3000 user profiles.

### Implementation details

The encoder of the VAE *f* consists of a fully connected layer with 256 neurons, while the dimension of the latent space (i.e.,$f_{\mu }$ and $f_{\sigma }$) is chosen equal to 256. The decoder of the VAE is a GRU that consists of 2 recurrent layers with 512 hidden units each. Regarding the target energy intake of Eq. 8, in our experiments, the parameter *D* is set to be equal to 500 calories based on feedback received from nutritional experts. Additionally, for all experiments, the GPT-3.5 model, updated on September 11, 2023, is employed. The method is trained with Adam optimizer with a learning rate of $10^{-4}$ and batch size of 64 for 500 epochs. During inference, the method samples 7 latent vectors for the decoder, in order to generate 7 daily meal plans and construct the weekly meal plan for the user. The generated meal plans of our proposed method are evaluated in terms of accuracy using mean and standard deviation. More specifically, the energy intake and nutrient (proteins, carbohydrates, fat and saturated fat) quantities are compared against the recommended nutritional targets^16^.

### Method evaluation

**Table 2:** Accuracy of the proposed diet recommendation method in the original PROTEIN NAP database (without ChatGPT utilization).

	Macronutrients accuracy					Caloric difference
	Average	Fat	SFA	Protein	Carbohydrates	Mean ± Std (%)
Healthy adults	89.66	88.74	90.59	–	–	0.0 ± 0.0
Healthy adolescents	88.97	87.52	90.42	–	–	0.0 ± 0.0
Healthy older adults	88.22	87.02	89.42	–	–	0.0 ± 0.0
Adults who are overweight	82.38	86.49	90.53	87.89	64.60	0.0 ± 0.0
Athletes	86.37	84.88	86.41	84.05	90.15	0.0 ± 0.0
Adults who are obese	89.60	85.08	91.68	88.48	93.16	0.0 ± 0.0
Adults with CVD	81.15	60.68	91.48	86.75	85.71	0.0 ± 0.0
Adults with T2D	86.58	84.24	86.59	86.73	82.30	0.0 ± 0.0
Adults with iron deficiency	87.48	87.14	87.82	–	–	0.0 ± 0.0
Adults with a diet low in fruits/vegetables	89.63	88.63	90.63	–	–	0.0 ± 0.0
Average	87.00	84.04	89.55	86.78	83.18	0.0 ± 0.0

This section aims to evaluate the effectiveness of the proposed method by evaluating it in terms of energy intake and macronutrient accuracy using the PROTEIN NAP database. The first series of experiments is conducted with the proposed method utilizing the optimizer and the results are presented in Table 2. The weekly meal plans are analyzed in terms of the accuracy of macronutrients i.e., Fat, SFA, Protein and Carbohydrates and the difference in calories (mean and standard deviation) is reported for each of the 10 user groups of the database. Overall, the system manages to achieve an average macronutrient accuracy of 87% proving that the suggested meals are nutritionally balanced and in accordance with the users’ needs. Furthermore, with the usage of the optimizer that adjusts the meal portions, the calories of the suggested meal plans have no deviation from the recommended energy intake on all user categories. This ensures that the proposed diets are personalized and match exactly the daily energy intake requirements of the users. The average macronutrient accuracy across all categories is highest for SFA (89.55%) and lowest for carbohydrates (83.18%), with an overall average macronutrient accuracy of 86.99%. The system has a satisfactory performance in terms of macronutrient accuracy for healthy adults and adults with a diet low in fruits and vegetables, both having an average accuracy above 89%. However, the system showed relatively lower accuracy for adults with CVD (81.16%) and adults with excess weight (82.34%). Despite some variations in macronutrient accuracy across different categories, the proposed system demonstrates high accuracy in providing diet recommendations tailored to the specific needs of each individual, successfully taking into consideration the anthropometric features and the medical condition of each individual.

Figure 5 illustrates the latent space of the VAE, by sampling the latent representations of the user profiles, as formed after the training of the proposed AI-based diet recommendation method, and then using the t-SNE^45^ dimensionality reduction technique to map the latent space to a two-dimensional space that can be plotted. From the visualization of the latent space, it is observed that users from the same population group are clustered together in the latent space, while clusters with similar characteristics are also close to each other. More specifically, the healthy population i.e., healthy adolescents, adults, older adults, and athletes, is depicted in neighbouring clusters in the bottom right of Fig. 5, while users with excess weight (i.e., overweight or obese) are depicted in clusters located in the upper middle part of Fig. 5. On the other hand, adults with medical conditions, such as CVD and T2D appear in neighbouring clusters in the middle of the latent space, bordering both healthy and overweight population groups, thus denoting that CVD and T2D users can also be healthy or overweight/obese. The visualization of the latent space reveals the ability of the proposed diet recommendation method to accurately model user profiles, effectively discriminating users based on their BMI, body composition and health factors. With the use of a novel generative network architecture and sophisticated loss functions, the proposed diet recommendation method can robustly group users in clusters in a rational way, proving that it can generalize to various user categories.

**Table 3:** Characteristics of 20 users used for testing the ChatGPT models.

User ID	Weight	Height	PAL	BMI	BMR	Age	Iron Deficiency	T2D	Heart disease	Subgroup
1	68	1.73	1.195	22.72	1596.83	41	No	No	No	Healthy adults
2	66	1.76	1.495	21.31	1516.31	53	No	No	No	Healthy adults
3	83	1.82	1.495	25.06	1982.90	16	No	No	No	Healthy adolescents
4	51	1.71	1.495	17.44	1384.00	15	No	No	No	Healthy adolescents
5	61	1.67	1.495	21.87	1147.99	88	No	No	No	Healthy older adults
6	149	1.82	1.495	44.98	2503.77	80	No	No	No	Healthy older adults
7	91	1.82	1.495	27.47	1953.83	40	No	No	No	Adults who are overweight
8	97	1.85	1.195	28.34	2025.90	44	No	No	No	Adults who are overweight
9	48	1.77	2.2	15.32	1309.90	30	No	No	No	Athletes
10	46	1.63	1.745	17.31	1256.69	28	No	No	No	Athletes
11	130	1.64	1.195	48.33	1980.25	41	No	No	No	Adults who are obese
12	112	1.64	1.195	41.64	1809.47	42	No	No	No	Adults who are obese
13	138	1.75	1.195	45.06	2544.22	41	Yes	No	Yes	Adults with CVD
14	180	1.82	1.195	54.34	3106.42	47	No	No	Yes	Adults with CVD
15	124	1.69	1.195	43.41	1944.58	40	Yes	Yes	No	Adults with T2D
16	66	1.76	1.195	21.31	1607.14	37	No	Yes	No	Adults with T2D
17	95	1.74	1.495	31.38	1735.21	30	Yes	No	No	Adults with iron deficiency
18	72	1.61	1.195	27.78	1425.97	43	Yes	No	No	Adults with iron deficiency
19	85	1.76	1.495	27.44	1867.36	36	No	No	No	Adults with low fruit & veg
20	97	1.82	1.195	29.28	2034.21	40	No	No	No	Adults with low fruit & veg

### Comparison with ChatGPT

Another experiment was conducted with a subset of 20 users, retrieved from the 3000 user profiles of the PROTEIN NAP database, with the goal to compare the proposed diet recommendation method, enhanced with knowledge on nutritional guidelines, against a ChatGPT-based recommender system. The subset of users, presented in Table 3, was created by randomly selecting two users from each of the 10 population groups. The use of a small subset of the original set of user profiles was necessitated due to the need for tasking ChatGPT to provide a weekly meal plan for each cuisine and for each individual user profile. The weekly meal plan recommendation is conducted by instructing ChatGPT to act as an intelligent system, which is skilled at generating personalized meal plans for four specified cuisines, i.e., International, Greek, Italian and Portuguese. Then, the user profile is used as query and ChatGPT generates the personalized weekly meal plan for the user. The results of this experiment are presented in Table 4, in which it can be deduced that the proposed method outperforms all ChatGPT-based recommenders, regardless of the specific cuisine used for the training of the method. In particular, the proposed method manages to achieve $0.00\pm 0.00$ caloric difference, thanks to the ability of the optimizer to adjust meal quantities so that the energy intake of the proposed meal plans matches exactly the suggested energy intake for the users. On the other hand, the difference between the energy intake of the ChatGPT generated meal plans and the target energy intake of users presents an average value of around $17\%$, with several generated meals containing $30\%$ more or less calories that the targeted values. Regarding macronutrient accuracy, the proposed method, enhanced with the equivalent meals from ChatGPT, manages to achieve an average accuracy improvement of almost $12\%$ with respect to ChatGPT recommendations, with the lowest difference appearing for the Portuguese cuisine ($2.85\%$) and the highest difference occuring for the Italian cuisine ($17.32\%$). These results demonstrate that ChatGPT provides meal plans of mediocre accuracy in terms of energy intake and nutritional content, while the proposed diet recommendation method can significantly improve the accuracy of the suggested meal plans due to the use of nutritional guidelines to align the method with the personalized dietary needs of the users.

**Figure 6:**
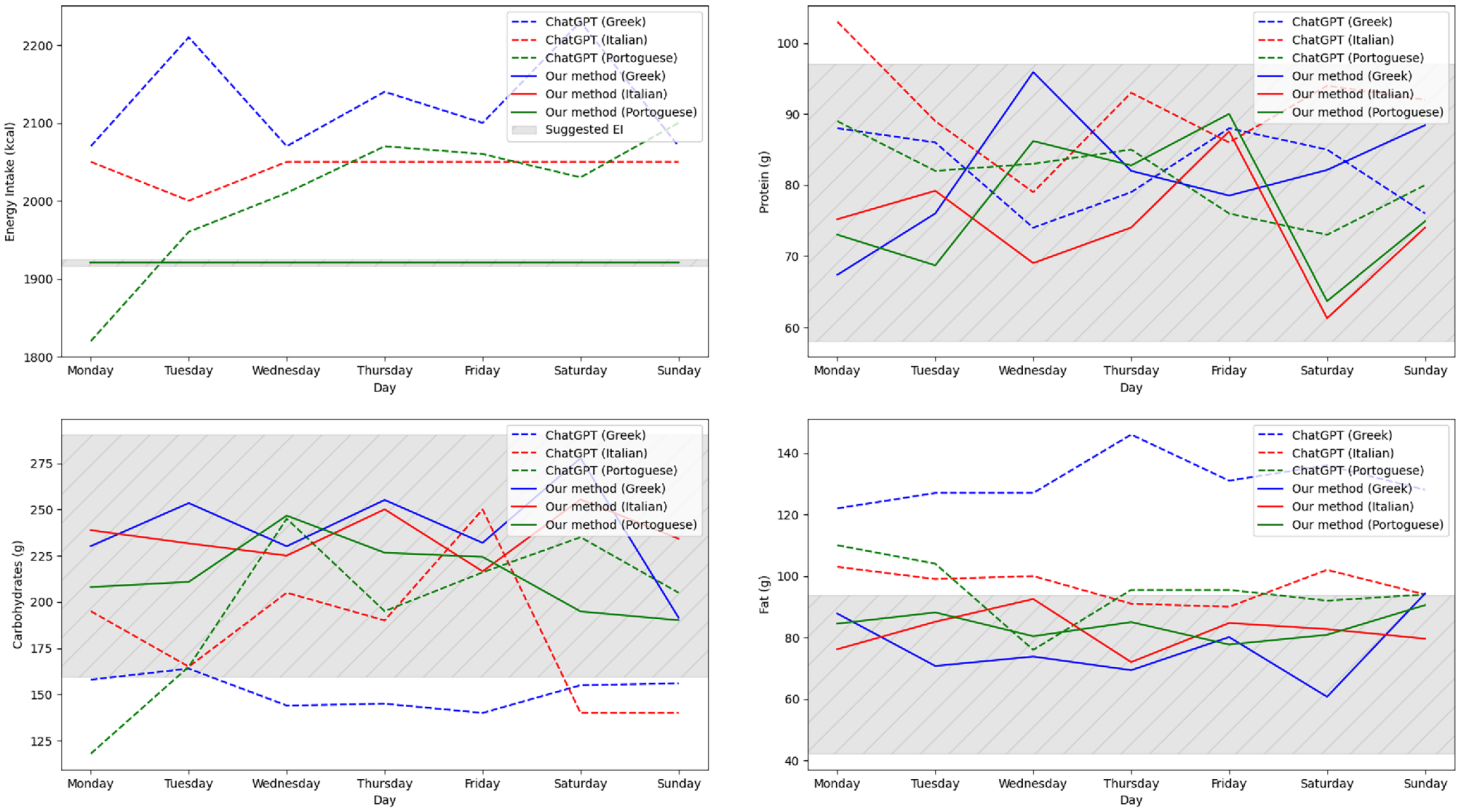
Weekly meal plan statistics for meal plans generated by ChatGPT and the proposed method for an obese patient. The grey zone represents the acceptable range of values for the respective nutrient (i.e., carbohydrates, protein, fat) based on the nutrition requirements of the user.

In Fig. 6, the daily caloric and macronutrient intake of a user with obesity (i.e., User 11), as recommended by ChatGPT and the proposed method for different cuisines, is depicted. It can be observed that ChatGPT recommends meal plans with higher caloric intake than the targeted one. On the contrary, the meal plans generated by the proposed method have the exact caloric content as estimated by Eq. 8 for all cuisines. Additionally, the macronutrient content of the meal plans generated by ChatGPT occasionally fall outside the designated daily target ranges, while showing strong fluctuations from day to day. Whereas, the proposed diet recommendation method suggests meal plans that consistently align with the desired macronutrient range of values and have steadier values from day to day. These results further verify the ability of the proposed AI-based diet recommendation method to generate highly accurate, nutritious and balanced meal plans, catered to the specific needs of each user, when distilled with knowledge on nutritional guidelines.

**Table 4:** Macronutrient and calorie accuracy of the weekly meal plans generated for 20 users.

	Caloric difference	Macronutrients accuracy
Method	Mean ± Std (%)	Mean (%)
ChatGPT (Greek)	17.18 ± 14.35	66.43
ChatGPT (Portuguese)	17.63 ± 15.28	74.29
ChatGPT (Italian)	18.31 ± 16.48	61.79
Our + ChatGPT (Greek)	0.00 ± 0.00	81.07
Our + ChatGPT (Portuguese)	0.00 ± 0.00	77.14
Our + ChatGPT (Italian)	0.00 ± 0.00	79.11

**Table 5:** Evaluation of the optimizer on the weekly meal plans generated for 20 users.

	Caloric difference	Macronutrients accuracy
Method	Mean ± Std (%)	Mean (%)
Our w/o optimizer (Protein NAP meals)	5.01 ± 3.87	84.18
Our w/o optimizer (Greek meals)	6.03 ± 4.48	80.18
Our w/o optimizer (Portuguese meals)	7.15 ± 5.23	76.23
Our w/o optimizer (Italian meals)	6.91 ± 5.43	77.56
Our w/ optimizer (Protein NAP meals)	0.00 ± 0.00	84.12
Our w/ optimizer (Greek meals)	0.00 ± 0.00	81.07
Our w/ optimizer (Portuguese meals)	0.00 ± 0.00	77.14
Our w/ optimizer (Italian meals)	0.00 ± 0.00	79.11

### Evaluation of optimizer

To assess the role of optimizer in the performance of the proposed meal recommendation system, additional experiments were performed on different cuisines with the addition or not of the optimizer. The results are presented in Table 5 and reveal the ability of the optimizer to adjust the meal quantities in a way that zeroes out the caloric differences of the meals with respect to the target energy intake values, while simultaneously retaining or even improving the macronutrient accuracy of the generated meal plans. More specifically, the use of the optimizer leads to an increase in the macronutrient accuracy of the generated meal plans by around $1\%$ for the Greek, Portuguese and Italian cuisine and a negligible decrease of $0.06\%$ in the macronutrient accuracy for the initial PROTEIN meal database. Furthermore, a comparison can be made between these results and the results of the ChatGPT model in Table 4 to demonstrate the ability of the proposed diet recommendation system to generate meal plans that are more accurate and balanced in terms of calories and macronutrients than those produced by ChatGPT either with or without the use of the optimizer. This means that the higher accuracy is mainly attributed to the better meal selection performed by the proposed system and is affected to some extent by the meal quantity adjustments performed by the optimizer.

### Results with equivalent meals

**Figure 7:**
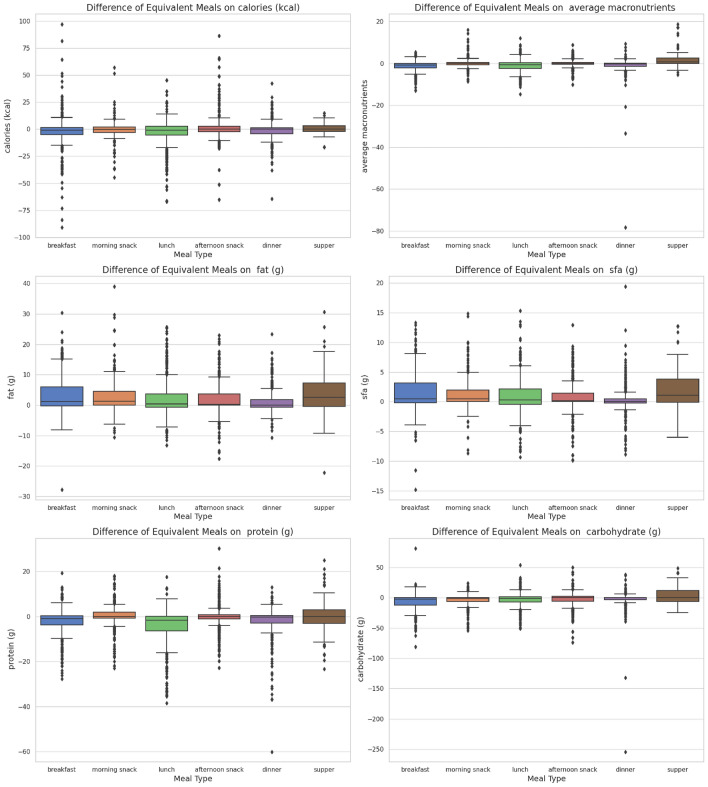
Visualization of the difference of energy and macronutrients of the equivalent meals.

Another experiment was conducted to evaluate the ability of ChatGPT to generate equivalent meals for other cuisines in terms of energy intake and macronutrient content. The importance of such an experiment lies in the need to expand the original meal database so that it can be applicable to different population groups. Figure 7 illustrates the differences in calories and macronutrients between the equivalent meals generated by ChatGPT and those in the Protein NAP database for each meal type. The whiskers, extending from the box represent where the majority of the generated meals reside. In general, it is observed that the majority of equivalent meals have similar nutritional and calorie characteristics with those in the original meal database for all meal types. More specifically, most meals have a caloric difference of less than 25 calories, while all meals have a difference of less than 100 calories, showcasing the ability of ChatGPT to generate equivalent meals in terms of energy intake. Similar conclusions can be drawn for the macronutrient difference between the equivalent meals as well, with mean and standard deviation differences being $-1.76 \pm 6.70$g for protein, $-3.56 \pm 12.64$g for carbohydrates, $2.29 \pm 5.19$g for fat and $0.97 \pm 2.74$g for SFA. Overall, the small variations in the equivalent meals prove that ChatGPT can successfully generate meals for other cuisines that align with the nutritional profile of the meals in the original PROTEIN NAP database. A few outliers that are plotted as dots with extreme values away from the box and whiskers reveal that ChatGPT can also produce inaccuracies that can be attributed to the lack of a one-to-one equivalence between foods in different cuisines. However, these outliers are small in number and do not hinder the ability of ChatGPT to expand a meal database to incorporate meals for other cuisines. This observation is further verified in Table 6 that depicts the statistical significance of the differences in energy intake and macronutrient content of the equivalent meals generated from ChatGPT. To this end, a statistical analysis of the meal content was performed and *p*-values are reported. As it can be seen, most *p*-values for the different cuisines and the types of macronutrients and energy intake are smaller than 0.001, revealing that the hypothesis of the meals being equivalent is of high statistical significance with 99.9% confidence. Slightly higher *p*-values are observed for the caloric difference of the meals in the PROTEIN NAP database and the equivalent Portuguese meals, as well as for the difference in protein between the meals in the PROTEIN NAP database and the equivalent Greek meals, but still these values are small enough (i.e., less than 0.05) to respect the hypothesis regarding the meal equivalence with a confidence higher than 95%.

**Table 6:** Statistical significance of energy intake and macronutrient content differences between equivalent meals.

Meals	*p*-value				
	Calories	Fat	Saturated fat (SFA)	Protein	Carbohydrates
Greek	< 0.001	< 0.001	< 0.001	0.0154	< 0.001
Italian	< 0.001	< 0.001	< 0.001	< 0.001	< 0.001
Portuguese	0.0018	< 0.001	< 0.001	< 0.001	< 0.001

Finally, the proposed diet recommendation engine was evaluated using the entire set of 3000 user profiles in its ability to generate accurate weekly meal plans for different cuisines by utilizing the equivalent meals generated by ChatGPT. The accuracy of the method was evaluated in terms of energy intake and macronutrient content with respect to the target values. The results, presented in Table 7, demonstrate the ability of the proposed diet recommendation method to generate meal plans with high accuracy for different cuisines, population groups and medical conditions. More specifically, for all cuisines the energy intake difference is zero due to the ability of the optimizer to adjust meal quantities based on the caloric content, while the average macronutrient accuracy is higher than 81% for all cuisines. A comparison among the different cuisines shows that the accuracy of the proposed diet recommendation method is only slightly affected by the different meals. This accuracy difference can be attributed to the small inaccuracies in the equivalent meals generated by ChatGPT. As a result, the proposed diet recommendation engine can successfully suggest weekly meal plans irrespective of the cuisine and the anthropometric features or the medical condition of the users.

**Table 7:** Macronutrient accuracy of weekly meal plans for 3000 users using equivalent meals from Greek, Italian and Portuguese cuisine.

	Caloric difference (%)	Macronutrients accuracy (%)				
Method	Mean ± Std	Average	Fat	SFA	Protein	Carbohydrates
Our + Protein NAP meals	0.00± 0.00	87.00	84.04	89.55	86.78	83.18
Our + Greek equivalent meals	0.00± 0.00	86.98	84.06	89.49	88.07	83.16
Our + Italian equivalent meals	0.00± 0.00	81.83	72.56	84.96	88.43	83.47
Our + Portuguese equivalent meals	0.00± 0.00	81.31	72.93	84.40	88.69	82.13

**Table 8:** Variability of recommended weekly meal plans.

Methods	Breakfast	Morning snack	Lunch	Afternoon snack	Dinner	Supper	Average
Proposed method (Greek)	4.73 ± 1.92	4.94 ± 1.89	4.78 ± 1.92	5.12 ± 1.88	4.79 ± 1.93	5.59 ± 1.80	4.99 ± 1.90
Proposed method (Italian)	4.72 ± 1.91	4.95 ± 1.89	4.79 ± 1.92	5.10 ± 1.88	4.80 ± 1.93	5.61 ± 1.81	4.98 ± 1.90
Proposed method (Portuguese)	4.66 ± 1.89	4.84 ± 1.86	4.70 ± 1.89	5.06 ± 1.86	4.76 ± 1.92	5.52 ± 1.80	4.88 ± 1.88

Apart from accuracy, meal variability is really important when generating a weekly meal plan since meal plans with repetitive dishes can lead to a loss of motivation for the user to adopt or follow the meal plans. In addition, meal variability ensures that the user consumes a wide range of nutrients and vitamins, resulting in a balanced and nutritious diet. In this study, we measure meal variability by assigning values ranging from 1 (a single daily meal plan is repeated throughout the week) to 7 (each daily meal plan is unique). The average meal variety of the proposed diet recommendation method is shown in Table 8. In general, the proposed method can achieve satisfying meal variability with an average value of 4.98 ± 1.90 for meals from the Protein NAP database, 4.99 ± 1.90 for Greek equivalent meals, 4.98 ± 1.88 for Italian equivalent meals and 4.88 ± 1.88 for Portuguese equivalent meals. This demonstrates that the proposed diet recommendation method is capable of generating weekly meal plans with high meal variability irrespective of the cuisine used, thus enhancing the attractiveness of the recommended meal plan for a user, facilitating the user adherence to the suggested nutritional advice.

### Results with real profiles

To demonstrate the ability of the proposed AI-based diet recommendation system to provide credible nutritional advice on humans, the 1000 real profiles collected from two datasets^43,44^ were fed to the trained system and 7000 daily meal plans from the International cuisine were generated. Afterwards, the accuracy of the generated meal plans in terms of energy intake and macronutrients was computed and the results are presented in Table 9. The evaluation conducted on human profiles reveals that the proposed diet recommendation system can generate meal plans with high accuracy for all user groups. More specifically, the average energy intake accuracy of the generated meal plans is 100%, while the average macronutrient accuracy is 84.19%. For all users groups, the average macronutrient accuracy is over 80%, with the lowest one observed for adults who are overweight (81.69%) and the highest one observed for adults who are obese (86.66%). These results demonstrate the usefulness of the proposed diet recommendation system in the real world and they can be attributed to the generative nature of the proposed system that is capable of creating a descriptive latent space that models different distributions of user profiles. As a result, the proposed system can generate accurate daily and weekly meal plans even for user profiles not seen before, i.e., not included in the training phase.

**Table 9:** Accuracy of the proposed diet recommendation method in the 1000 real profiles.

	Macronutrients accuracy					Caloric difference
	Average	Fat	SFA	Protein	Carbohydrates	Mean ± Std (%)
Healthy adults	86.4	84.97	87.83	–	–	0.0 ± 0.0
Adults who are overweight	81.69	83.2	88.17	84.34	71.03	0.0 ± 0.0
Adults who are obese	86.66	85.03	91.6	85.43	84.57	0.0 ± 0.0
Adults with CVD	82.01	65.14	93.66	84.74	84.51	0.0 ± 0.0
Average	84.19	79.59	90.32	84.84	80.04	0.0 ± 0.0

## Discussion

In this study, a novel AI-based approach for personalized nutrition recommendation is introduced and evaluated. The proposed method utilizes a variational autoencoder to model user profiles in a descriptive latent space and a recurrent neural network to generate weekly meal plans, tailored to the needs of users. In addition, an optimizer is introduced to adjust meal quantities to ensure that the energy requirements of users are fulfilled. Finally, ChatGPT is adopted to expand the original meal database and enhance meal recommendations. The proposed nutrition recommendation system is aimed as a proof of concept for the generation of accurate personalized meal plans, with additional features and considerations (e.g., complex dietary needs and multiple food allergies) being added as a future work and during integration with applications. The conducted experiments demonstrated the effectiveness of the proposed diet recommendation method, indicating its potential to overcome challenges associated with AI alignment in the context of nutrition and diet planning. With the modelling of user profiles in a latent space through a deep generative network architecture, the proposed method is able to cluster users in groups with similar characteristics, even in challenging situations, in which users with various health conditions are involved (Fig. 5). This indicates the ability of the proposed method to take into consideration an array of measurements and health indicators to provide rational nutritional advice to users of different background and/or medical condition.

Moreover, the novel loss functions, as well as the proposed optimizer, guides the method towards producing highly accurate and personalized meal plans that satisfy the user’s suggested energy intake and macronutrient values, according to nutritional guidelines set by organizations, such as EFSA and WHO. A sample daily meal plan, with 2043 kcal Calories, 85.11g of Protein, 279.25g of Carbohydrates, 65.47g of Fat and 13.16g of SFA, generated by the proposed nutrition recommendation system can be found in Table 10. With loss terms that guide the network training towards well-founded nutritional guidelines and adjustments in meal quantities, the proposed method demonstrates zero caloric difference and highly accurate macronutrient accuracy (i.e., 87% in average for the 3000 virtual user profiles and 84.19% for the 1000 real profiles) with respect to the targeted values for different population groups (Tables 2 and 9). A comparison with ChatGPT-based diet recommendations reveals a few inaccuracies of ChatGPT (i.e., deviations in terms of macronutrients and calories) that are significantly diminished by the proposed method, thus justifying the need for distilling expert’s knowledge into AI systems to improve their accuracy and compliance with user needs (Table 4). Additionally, the visualization of weekly meal plan statistics in Fig. 6 demonstrates the ability of the proposed method to provide balanced daily meal plans with no significant fluctuations in energy and macronutrients during the week, in contrast to ChatGPT, where larger daily fluctuations are noticed. As a result, these experiments verify that the proposed method can effectively bridge the advantages of both AI and knowledge-based systems, achieving real-time inference speed (approximately 500 milliseconds to generate a weekly meal plan and significantly less than rule-based and ontology-based systems), low complexity (no need for elaborate ontologies) and high knowledge awareness (nutritional guidelines added as loss terms). In this way, the proposed method is capable of generating accurate, balanced and nutritious meal plans, aligned with the user’s dietary needs and health objectives to promote users’ overall health and well-being.

Furthermore, this study investigated the ability of ChatGPT to generate equivalent meal plans for different cuisines. As it was shown in Fig. 7, ChatGPT can achieve really high performance in producing meal equivalents. The proposed method leverages the ability of ChatGPT to search the web and identify an almost infinite pool of meals to expand the original PROTEIN NAP database with equivalent meals in other cuisines. In this way, an important limitation of current knowledge-based diet recommendation systems, whose accuracy and applicability is severely affected by small meal databases, is addressed. With the addition of new meal options that are equivalent with the validated meals, proposed by nutritional experts, of the PROTEIN NAP database, the proposed method can significantly enhance the variety of the generated meal plans, enhance user satisfaction and gastronomic experience and improve its applicability to other cuisines, while remaining aligned with users’ personalized nutritional needs as verified by the high accuracy of the proposed method irrespective of the cuisine employed in Table 7.

Besides the real-time performance, low complexity, compliance with nutritional guidelines, generalization ability and accuracy in terms of calories and macronutrients of the proposed method, it is important to note that the method is designed to be easily integrated into existing platforms, in contrast to knowledge-based methods, whose ontologies pose challenges to any integration attempt. As a result, the proposed method can be incorporated into applications, such as healthcare applications, fitness apps or dietary consulting services to be used in real-life scenarios to improve the health and well-being of users, while promoting nutritious and balanced diets. With the proposed method, users can have access to personalized weekly meal plans to achieve their own dietary goals, such as muscle gain or weight loss. In addition, users with health conditions, such as CVD and T2D, can leverage the provided personalized nutritional advice of the proposed method to prevent or cope with the symptoms of their diseases and improve their quality of life.

Another important aspect of the proposed method lies in the need for human evaluation to identify whether user preferences are covered by the proposed approach and whether the users can adopt the suggested diets and follow the recommended meal plans. To achieve this, the next step of this research work concerns the conduction of a study with real users and the collection and analysis of their opinions and considerations to further support and enhance the applicability of the proposed method.

**Table 10:** A sample daily meal plan generated by the proposed nutrition recommendation system.

Meal	Description
Breakfast	2 slices (60g each) of Bread with 1 portion (56g) of Cheese
Morning Snack	1 cup (240g) of Greek Yogurt with 1 portion (28g) of Nuts
Lunch	1 medium portion (113g) of Chicken, 1 cup (195g) of cooked Rice and 1 cup (150g) of Vegetables
Afternoon Snack	1 medium-sized (130g) Apple and 2 tablespoons (32g) of Peanut Butter
Dinner	Omelette with 3 Eggs (50g each), 1 cup (mixed, 100g) of Salad and 1 cup (150g) of Vegetables
Supper	1 cup (about 240g) of Soya Yoghurt

## Conclusion

This study introduced an AI-based diet recommendation method that combines deep generative networks with LLMs, such as ChatGPT to provide accurate personalized nutrition advice in the form of weekly meal plans. Utilizing the expressiveness of variational autoencoders to robustly model user features, such as anthropometric measurements and medical condition, into a highly descriptive latent space, as well as sophisticated loss functions to force the deep network to abide by well-established EFSA and WHO nutritional guidelines, the proposed method can significantly improve the explainability and accuracy of the generated diet recommendations. Additionally, the use of ChatGPT to expand the original meal database with equivalent meals from other cuisines leads to increased meal variety and improved accuracy, while enhances the applicability of the proposed method on different population groups. As a future work, the aim is to expand the knowledge base of the proposed method to incorporate a variety of international cuisines and regional dietary preferences. In addition, to accommodate particular dietary needs, such as vegan and gluten-free or specific allergies, the proposed method will in future take into consideration specific dietary guidelines to provide accurate recommendations to users with specific dietary preferences. Finally, a study with real users is needed to assess user satisfaction and adoption of the recommendation meal plans.

## Data Availability

The datasets used and/or analysed during the current study are available from the corresponding author on reasonable request.
